# Evaluation of novel coronavirus disease (COVID-19) using quantitative lung CT and clinical data: prediction of short-term outcome

**DOI:** 10.1186/s41747-020-00167-0

**Published:** 2020-06-26

**Authors:** João Matos, Francesco Paparo, Ilaria Mussetto, Lorenzo Bacigalupo, Alessio Veneziano, Silvia Perugin Bernardi, Ennio Biscaldi, Enrico Melani, Giancarlo Antonucci, Paolo Cremonesi, Marco Lattuada, Alberto Pilotto, Emanuele Pontali, Gian Andrea Rollandi

**Affiliations:** 1 DISSAL—Department of Health Sciences, University of Genoa, Via Antonio Pastore, 1, 16132 Genova, GE Italy; 2 Department of Radiology, Galliera Hospital, Genoa, Italy; 3 Independent Researcher, Plymouth, UK; 4 Department of Critical Care Medicine, Galliera Hospital, Genoa, Italy; 5 Department of Emergency Medicine, Galliera Hospital, Genoa, Italy; 6 Department of Anesthesiology, Galliera Hospital, Genoa, Italy; 7 Department of Geriatric Medicine, Galliera Hospital, Genoa, Italy; 8 Department of Infectious Diseases, Galliera Hospital, Genoa, Italy

**Keywords:** COVID-19, Lung, SARS-Cov-2, Pneumonia (viral), Support vector machine, Tomography (x-ray computed)

## Abstract

**Background:**

Computed tomography (CT) enables quantification of severe acute respiratory syndrome coronavirus 2 (SARS-CoV-2) infection, helping in outcome prediction.

**Methods:**

From 1 to 22 March 2020, patients with pneumonia symptoms, positive lung CT scan, and confirmed SARS-CoV-2 on reverse transcription-polymerase chain reaction (RT-PCR) were consecutively enrolled. Clinical data was collected. Outcome was defined as favourable or adverse (*i.e.*, need for mechanical ventilation or death) and registered over a period of 10 days following CT. Volume of disease (VoD) on CT was calculated semi-automatically. Multiple linear regression was used to predict VoD by clinical/laboratory data. To predict outcome, important features were selected using a priori analysis and subsequently used to train 4 different models.

**Results:**

A total of 106 consecutive patients were enrolled (median age 63.5 years, range 26–95 years; 41/106 women, 38.7%). Median duration of symptoms and C-reactive protein (CRP) was 5 days (range 1–30) and 4.94 mg/L (range 0.1–28.3), respectively. Median VoD was 249.5 cm^3^ (range 9.9–1505) and was predicted by lymphocyte percentage (*p* = 0.008) and CRP (*p* < 0.001). Important variables for outcome prediction included CRP (area under the curve [AUC] 0.77), VoD (AUC 0.75), age (AUC 0.72), lymphocyte percentage (AUC 0.70), coronary calcification (AUC 0.68), and presence of comorbidities (AUC 0.66). Support vector machine had the best performance in outcome prediction, yielding an AUC of 0.92.

**Conclusions:**

Measuring the VoD using a simple CT post-processing tool estimates SARS-CoV-2 burden. CT and clinical data together enable accurate prediction of short-term clinical outcome.

## Key points

- Volume of disease (VoD) on computed tomography (CT) scan and clinical information predict early outcome in COVID-19 patients.
- VoD on CT scan was predicted by lymphocyte percentage and C-reactive protein.
- CT may help in guiding clinical management of COVID-19 patients.

## Background

During the last weeks of 2019, a previously unknown virus of the *Coronaviridae* family acquired the capability of person-to-person transmission. The newly identified virus, designated severe acute respiratory syndrome coronavirus 2 (SARS-CoV-2), causes the coronavirus disease 2019 (COVID-19) [1]. In March 2020, it was declared a pandemic by the World Health Organization [2].

SARS-CoV-2 has the potential to cause a complex disease that includes severe pneumonia in some individuals. As the virus spreads in the population, health systems are pushed to their limits. The diagnosis of COVID-19 is made with reverse transcription-polymerase chain reaction (RT-PCR), mainly using nasopharyngeal swabs. This technique has some limitations. It has a suboptimal sensitivity, and results may not be readily available. In times when many patients seek medical attention due to symptoms suggestive of COVID-19, early detection of the disease plays a pivotal role for the correct isolation and treatment of patients with SARS-CoV-2 [3, 4].

Computed tomography (CT) has been shown to have high sensitivity for SARS-CoV-2 diagnosis in patients with respiratory symptoms [4–6]. It has been used with success for grading and follow-up of SARS-CoV-2 [7–10]. Furthermore, CT demonstrated promising results in predicting adverse outcomes in COVID-19 patients [11–13]. CT allows the extraction of many features ascribed to both COVID-19 and the patient’s underlying diseases. Previous works used qualitative and semi-quantitative CT-derived features to predict outcomes in COVID-19 [11, 12]. A recent work by Colombi et al. [13] concluded that well-aerated lung volume on admission CT scan could be used to predict short-term outcomes in COVID-19 patients. It is conceivable that the quantification of SARS-CoV-2 lung involvement, in the presence of other ancillary features, may help to identify patients that will have a severe disease course.

This study aimed to analyse the performance of combining quantitative CT with clinical and laboratory data to predict which patients are at risk of adverse clinical outcomes.

## Methods

This single-centre study was approved by the Institutional Review Board and written informed consent regarding the disclosure of personal data was obtained from all participants.

### Patient enrollment and clinical information

**Fig. 1:**
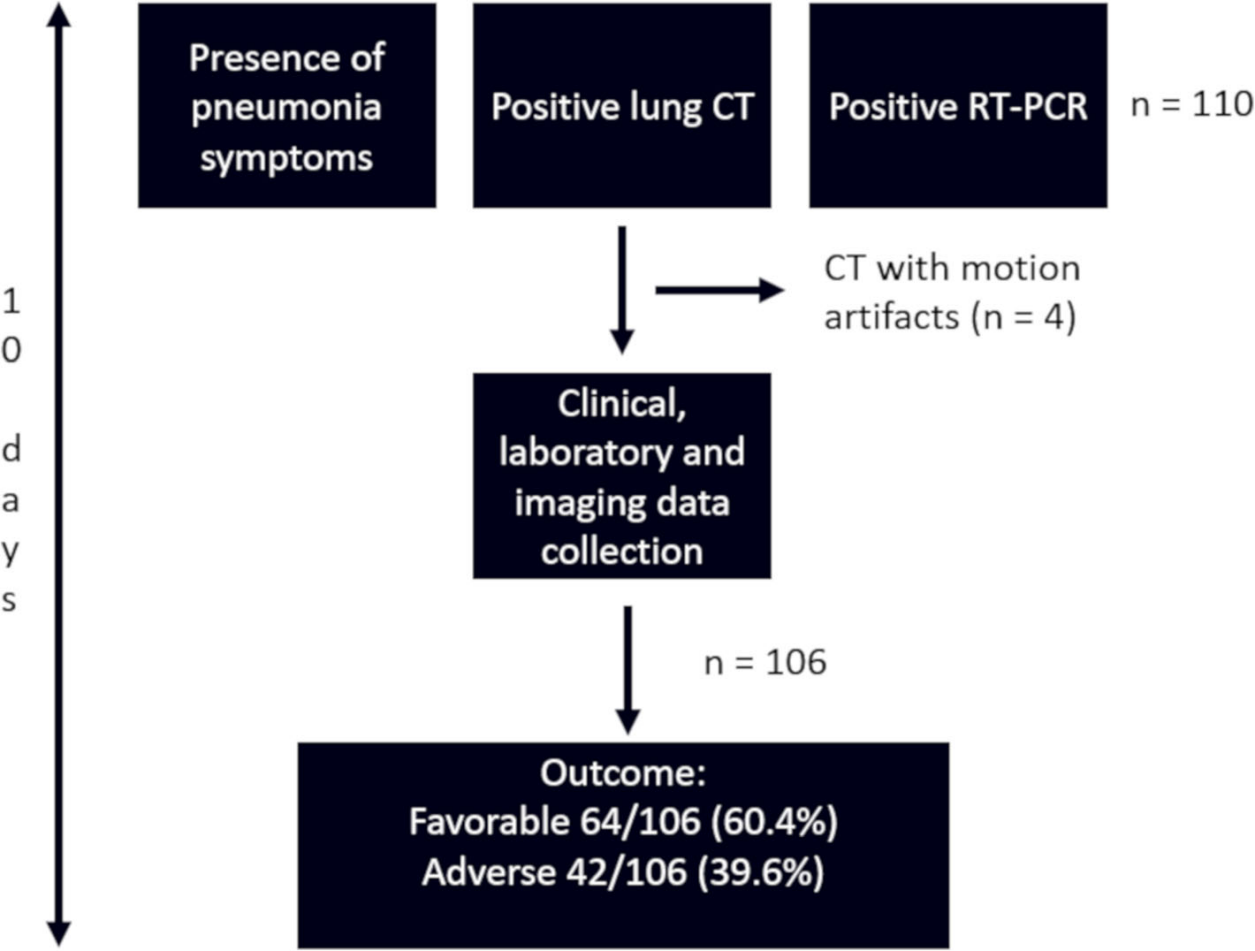
Flow chart diagram of the study design. CT, Computed tomography; RT-PCR, Reverse transcription-polymerase chain reaction

From 1 to 22 March of 2020, patients who (1) presented with pneumonia symptoms (two or more of the following: T ≥ 37.5 °C, cough, dyspnea), (2) had a positive lung CT scan, and (3) had confirmed SARS-CoV-2 infection on RT-PCR were consecutively enrolled. The timespan of enrollment coincided with the ascending phase of the pandemic in our region. We excluded patients with significant motion artifacts on CT scan, *i.e.*, respiratory artifacts that were present in the pulmonary bases plus other lung zones. A flow chart diagram is shown in Fig. 1.

We collected the following patient data: demographics (age, gender), clinical information (history of the present illness, duration of symptoms at time of CT scan), and comorbidities (oncologic disease, diabetes, end-stage kidney disease, or ongoing immunosuppressive therapy). Laboratory values (white blood cell count, lymphocyte percentage, and C-reactive protein serum levels) were obtained on the same day of the lung CT. Other relevant data (*e.g.,* D-dimer, erythrocyte sedimentation rate, peripheral capillary oxygen saturation) were not available in the clinical records of all patients and thus not collected for this study. We observed the clinical outcome over a period of 10 days following lung CT scan. Favourable outcome was specified as survival with or without need for supplemental oxygen therapy, excluding mechanical ventilation. Adverse outcome was defined as the need for mechanical ventilation or death.

### CT protocol

All patients underwent unenhanced CT with a 16-slice CT scanner (CT lightspeed-16, General Electric Healthcare, Chicago, IL, USA) when they came to clinical attention due to pneumonia symptoms. Radiation exposure was adapted to each patient’s body habitus. All CT examinations were performed with the patient in the supine position during a deep inspiration breath-hold. All patients wore a surgical mask except those who were undergoing oxygen therapy. CT parameters were as follows: tube voltage 120 kVp, smart mA tube current modulation (range 100–400 mA), NOISE index 13.88, pitch 1.75:1, and table movement 35 mm/rotation. Reconstructions were made with adaptive statistical iterative reconstruction with a 40% value at a slice thickness of 1.25 mm. After each examination, we performed surface disinfection with 62–71% ethanol or 0.1% sodium hypochlorite and passive air exchange was performed for 30–60 min.

### Image interpretation

**Fig. 2:**
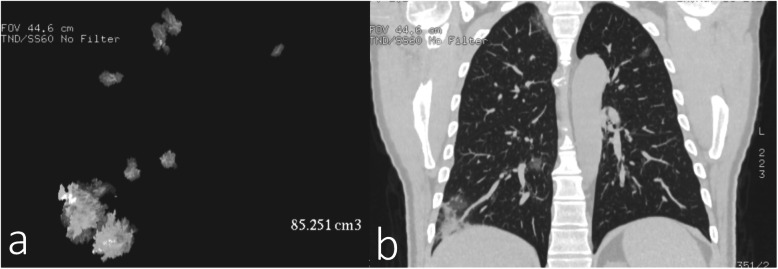
Example of severe acute respiratory syndrome coronavirus 2 lung disease segmentation. **a** Maximum intensity projection coronal image shows segmented lung opacities and the volume provided in cubic centimeters. **b** Corresponding coronal computed tomography image

**Fig. 3:**
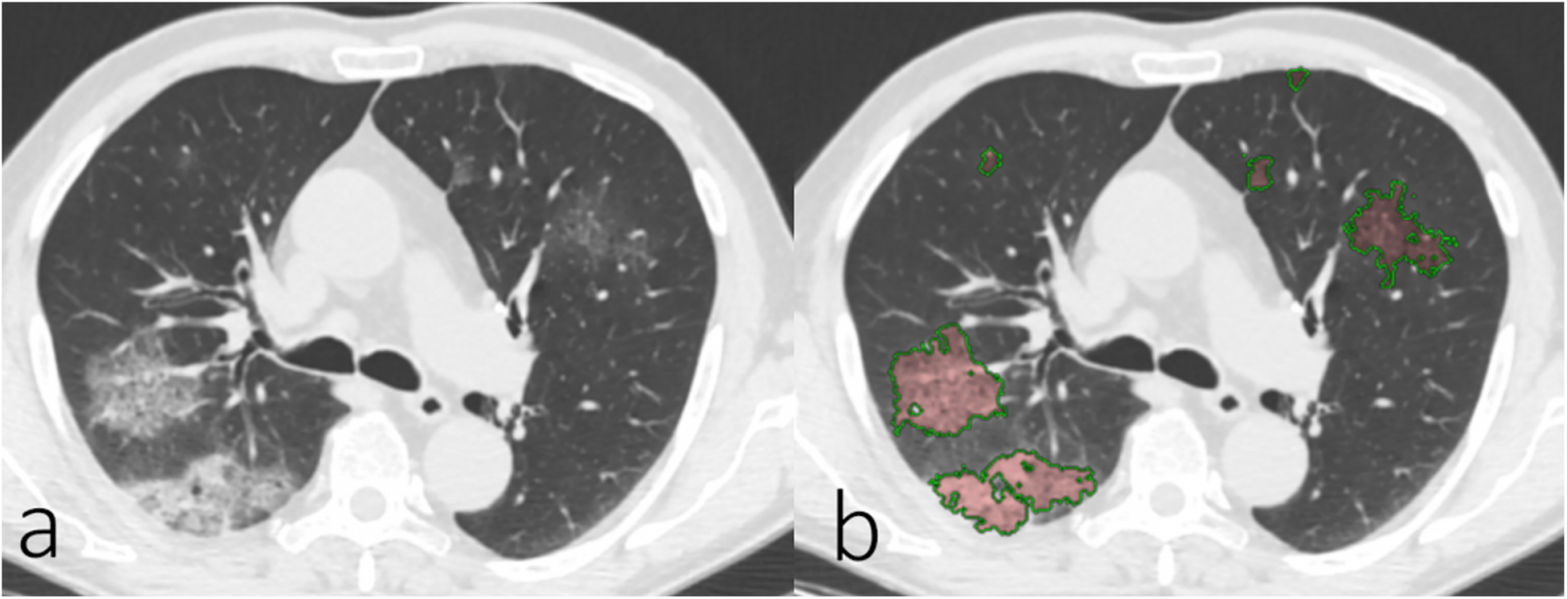
Example of severe acute respiratory syndrome coronavirus 2 (SARS-CoV-2) lung disease segmentation. Typical SARS-CoV-2 pneumonia with lung opacities before (**a**) and after (**b**) semiautomatic segmentation

Images were reviewed and processed in an AW Volumeshare 4 workstation (General Electric Healthcare, Chicago, IL, USA) by 2 different radiologists, F.P. and L.B., with 10 and 15 years of experience in thoracic imaging, respectively. The radiologists were blinded to the clinical and laboratory data. Volume of disease (VoD) was extracted using the “autoselect” function and the result was expressed in cubic centimeters. The radiologist selected the desired opacity and voxels with similar pixel values were automatically extracted (region growing). Corrections had to be made in cases of pulmonary consolidations adjacent to the chest wall or mediastinum. The duration of the segmentation task ranged from 2 to 5 min. Figures 2 and 3 depict segmentation examples. The remaining CT-derived data was obtained and expressed using a slightly modified version of the descriptive system used by Inui et al. [14]: involved lungs, gradient, distribution of disease, CT pattern type, predominant type of opacity, reverse halo, linear opacities, and nodules. We also registered the following secondary findings: enlarged thoracic lymph nodes, presence of pleural effusion or thickening, coronary and/or aortic calcification, chronic lung disease (emphysema or fibrosis), and other significant findings (such as pneumothorax or pneumomediastinum).

### Statistical analysis

Statistical analyses were performed using MedCalc for Windows, version 15.0 (MedCalc Software, Ostend, Belgium) and in the R statistical environment using Rstudio for Windows, version 1.2.335 (RStudio, Inc., Boston, MA, USA). Descriptive statistics were produced for demographic, clinical, laboratory, and CT imaging features of patients. Categorical data were expressed as number and percentage, while continuous data as median and range. The normal distribution of different datasets was assessed by means of the D’Agostino-Pearson test [15].

### Prediction of volume of disease by clinical and laboratory information

We assessed the prediction of VoD by clinical and laboratory data employing multiple linear regression analysis. We introduced the VoD as the dependent variable and the duration of symptoms, white blood cell count, percentage of lymphocytes, and serum levels of C-reactive protein as independent variables.

### Outcome prediction: variable importance a priori

We used the R package “caret” [16] to select important variables and to train predictive models, and the package “pROC” [17] to compute receiver operating characteristic (ROC) curves, which we used as diagnostic of model performance.

Our analysis aimed to test the performance of CT-derived, clinical, and laboratory information in predicting the outcome of SARS-CoV-2 infection using classification models. The outcome was divided into 2 classes, favourable outcome (survival) and adverse outcome (*i.e.,* need for mechanical ventilation or death), and used as response variable. The predictor variables included demographic, clinical, laboratory, and CT-derived information of patients. The predictors initially considered for analysis were gender (categorical, binomial), age (continuous, years), duration of symptoms (continuous, days), white blood cell count (continuous, 10^9^/L), lymphocyte percentage (continuous, expressed as percentage), serum levels of C-reactive protein (continuous, concentration in mg/L), VoD (continuous, cm^3^), predominant opacity type (categorical, binomial, defined as ground-glass opacities [GGO] or consolidation), chronic lung disease (categorical, trinomial, defined as no emphysema or fibrosis, predominant emphysema or predominant fibrosis), coronary calcification (categorical, binomial, no or yes), aortic calcification (categorical, binomial, no or yes), and presence of chronic comorbidity (categorical, binomial, no or yes).

We performed an a priori analysis to determine the variables more likely to predict the clinical outcome linked to SARS-CoV-2 infection. This selection was necessary to reduce the dimensionality of the dataset, thus avoiding overfitting and consequent loss of accuracy [18] as well as allowing for an easier interpretation and applicability of the results. The importance of predictors was evaluated individually through a filter-based method based on a ROC curve analysis. We applied a series of cutoffs to each predictor and calculated sensitivity and specificity in predicting the outcome. Sensitivity and specificity were used to build a ROC curve for each predictor. The method follows Kuhn and Johnson [19]. The area under the curve (AUC) was used as a measurement of variable importance. The AUC ranges from 0 to 1, where values equal or below 0.5 indicate variables predicting the response randomly. We selected only those variables whose AUC was equal or higher than the average AUC (0.65), a threshold high enough to ensure excluding variables too close to random predictive power (AUC of 0.5). All other variables were discarded.

### Outcome prediction: model training and testing

To find an optimal predictive model of SARS-CoV-2 outcome, we explored four classification algorithms. Generalised linear model (GLM) generalises the ordinary linear regression method when the error distribution of the response variable is non-normally distributed [20]. In our dataset, the response variable is binary; therefore, the GLM reduces to a logistic regression. Penalised binomial regression (PBR) is a type of logistic regression where the coefficients of the least predictive variables are shrunk toward zero [21], which favours simpler models and avoids overfitting. The conditional inference trees (CIT) algorithm recursively partitions the predictors to find a hierarchical tree structure predictive of the response variable [22]. The support vector machine with linear kernel (SVL) searches for linear decision boundaries that have maximum distance from the data points of all the classes indicated in the response variable [23].

The data was split between a training set (75% of the dataset, 80 patients) and a testing set (25% of the dataset, 26 patients). The training set was used to tune the parameters of the algorithms used for building the model and to generate the final model based on those parameters. All algorithms have tunable parameters except for GLM, which did not need training. Training was performed by iterating the algorithm using different parameter values and fivefold cross-validation, using one of the five folds to validate the performance of the training. The whole operation was repeated ten times, each time using a different fivefold resampling of the data, and the performance was measured by the AUC averaged across the five cross-validations and ten repetitions. The parameters yielding the highest AUC were chosen and the algorithms re-trained accordingly, without performing cross-validation, to obtain the final models. From each model, we extracted the variable contributions to the output.

The testing set provided data for estimating the performance of the built models. The performance was measured using accuracy (proportion of correctly predicted values), sensitivity (proportion of correctly identified positives), specificity (proportion of correctly identified negatives), positive predictive value (proportion of true positives over total positives), and negative predictive value (proportions of true negatives over total negatives), and AUC (area under the ROC curve), but only AUC was ultimately used to choose the best model.

## Results

### Clinical and laboratory information

A total of 106 patients met the inclusion criteria (median age 63.5 years, range 26–95, 41/106 women, 38.7%). Of 106 patients, 40 (37.7%) had at least one comorbidity. Median duration of symptoms and C-reactive protein levels at the time of CT scan were respectively 5 days (range 1–30) and 4.94 mg/L (range 0.1–28.3). Ninety-seven of 106 (91.5%) patients were admitted and 9/106 were discharged from the emergency department. Of 106 patients, 64 (60.4%) had a favourable outcome, and 42 (39.6%) had an adverse outcome (need for mechanical ventilation or death). Table 1 illustrates demographic, clinical, and laboratory data of the study population.

**Table 1:** Demographic, clinical, and laboratory data of the study population

Demographics	
Age (years; median, range)	63.5 (26–95)
Male (number, percentage)	65/106 (61.3)
Female (number, percentage)	41/106 (38.7)
Clinical information	
No comorbidity (number, percentage)	66/106 (62.3)
Presence of ≥ 1 comorbidity	40/106 (37.7)
Duration of symptoms at computed tomography (days; median, range)	5 (1–30)
Laboratory information	
White blood cell count (10^9^/L) (median, range)	5.7 (1.9–29.7)
Lymphocyte (%) (median, range)	18.8 (2.2–53.0)
C-reactive protein (mg/L) (median, range)	4.94 (0.1–28.3)
Admitted/discharged from emergency department	
Admitted	97/106 (91.5%)
Discharged from the emergency department	9/106 (8.5%)
Outcome	
Favourable	64/106 (60.4%)
Adverse	42/106 (39.6%)
Outcome subgroups	
Need for mechanical ventilation	17/42 (40.5%)
Death	25/42 (59.5%)

### CT-derived data

Median VoD caused by SARS-CoV-2 was 249.5 cm^3^ (range 9.9–1505). The disease was bilateral in 99/106 patients (93.4%) and was present in the lower lobe(s) and at least in another lobe in 97/106 (91.5%). In 65/106 (61.3%) of cases both peripheral and central lung regions were affected. The most common CT pattern was GGO in association with consolidation in 49/106 (46.2%), with GGO being predominant over consolidation in 79/106 (74.5%) of cases. The most prevalent CT sign were linear opacities in 66/106 (62.3%). The most common secondary finding was coronary calcifications in 53/106 (50.0%). Table 2 shows all CT findings.

**Table 2:** Quantitative and qualitative computed tomography (CT) findings related to COVID-19, and secondary CT findings

Volume of disease (cm^3^; median, range)	249.5 (9.9–1505)
Uni/bilateral	
Unilateral	7/106 (6.6%)
Bilateral	99/106 (93.4%)
Affected lobes	
Only lower lobe(s)	5/106 (4.7%)
Lower lobe(s) + at least one other lobe	97/106 (91.5%)
No lower lobe involvement	4/106 (3.8%)
Gradient	
Apicobasal gradient	49/106 (46.2%)
No apicobasal gradient	57/106 (53.8%)
Distribution	
Peripheral	39/106 (36.8%)
Central	2/106 (1.9%)
Mixed	65/106 (61.3%)
CT pattern	
Pure GGO	11/106 (10.4%)
GGO + septal thickening	46/106 (43.4%)
GGO + consolidation	49/106 (46.2%)
Predominant type	
GGO	79/106 (74.5%)
Consolidation	27/106 (25.5%)
CT sign	
Reverse halo	7/106 (6.6%)
Linear opacities	66/106 (62.3%)
Nodules	28/106 (26.4%)
Secondary findings	
Emphysema	13/106 (12.3%)
Fibrosis	8/106 (7.5%)
Enlarged lymph nodes (≥ 10 mm short axis)	33/106 (31.1%)
Pleural effusion	10/106 (9.4%)
Pleural thickening	15/106 (14.15%)
Aortic Calcification	45/106 (42.5%)
Coronary calcification	53/106 (50.0%)
Other	
Pneumomediastinum	1/106 (0.9%)
Iatrogenic pneumothorax and subcutaneous emphysema	1/106 (0.9%)

*GGO* Ground-glass opacities

### Volume of disease prediction by clinically derived data

VoD was predicted by lymphocyte percentage (*p* = 0.008) and C-reactive protein levels (*p* < 0.001). Duration of symptoms (*p* = 0.184) and white blood cell count (*p* = 0.229) did not significantly predict VoD.

### Variable selection through a priori analysis of variable importance

**Fig. 4:**
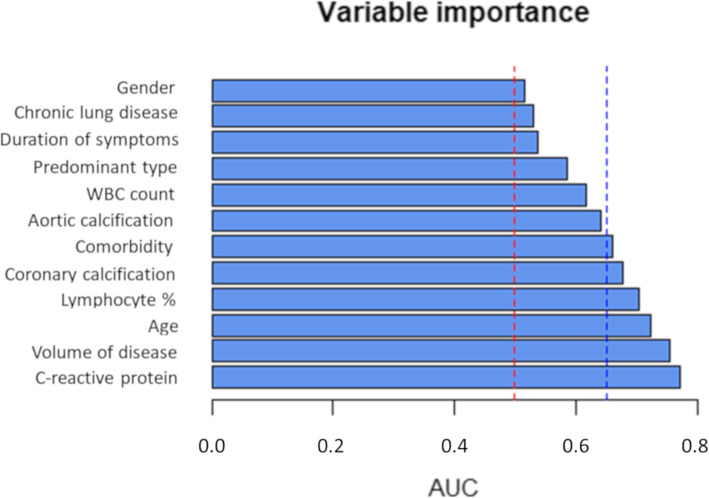
A priori analysis for variable selection. The red line is set at area under the curve (AUC) value below 0.5, below which variables predict the response randomly. The blue line is set at AUC value of 0.65. Variables to the right of this line are above a threshold high enough to ensure strong predictive power. WBC*,* White blood cell count

The a priori analysis of variable importance yielded unsatisfactory AUCs (below 0.65) for six of the variables tested, namely aortic calcification (AUC 0.64), white blood cell count (AUC 0.62), predominant opacity type (AUC 0.58), duration of symptoms (AUC 0.54), chronic lung disease (AUC 0.53), and sex (AUC 0.52). The six remainder variables were selected for inclusion in the predictive models: serum levels of C-reactive protein (AUC 0.77), VoD (AUC 0.75), age (AUC 0.72), lymphocyte percentage (AUC 0.70), coronary calcifications (AUC 0.68), and presence of chronic comorbidity (AUC 0.66). Figure 4 shows the AUC for all the tested variables.

### Outcome prediction by CT, clinical, and laboratory information

**Fig. 5:**
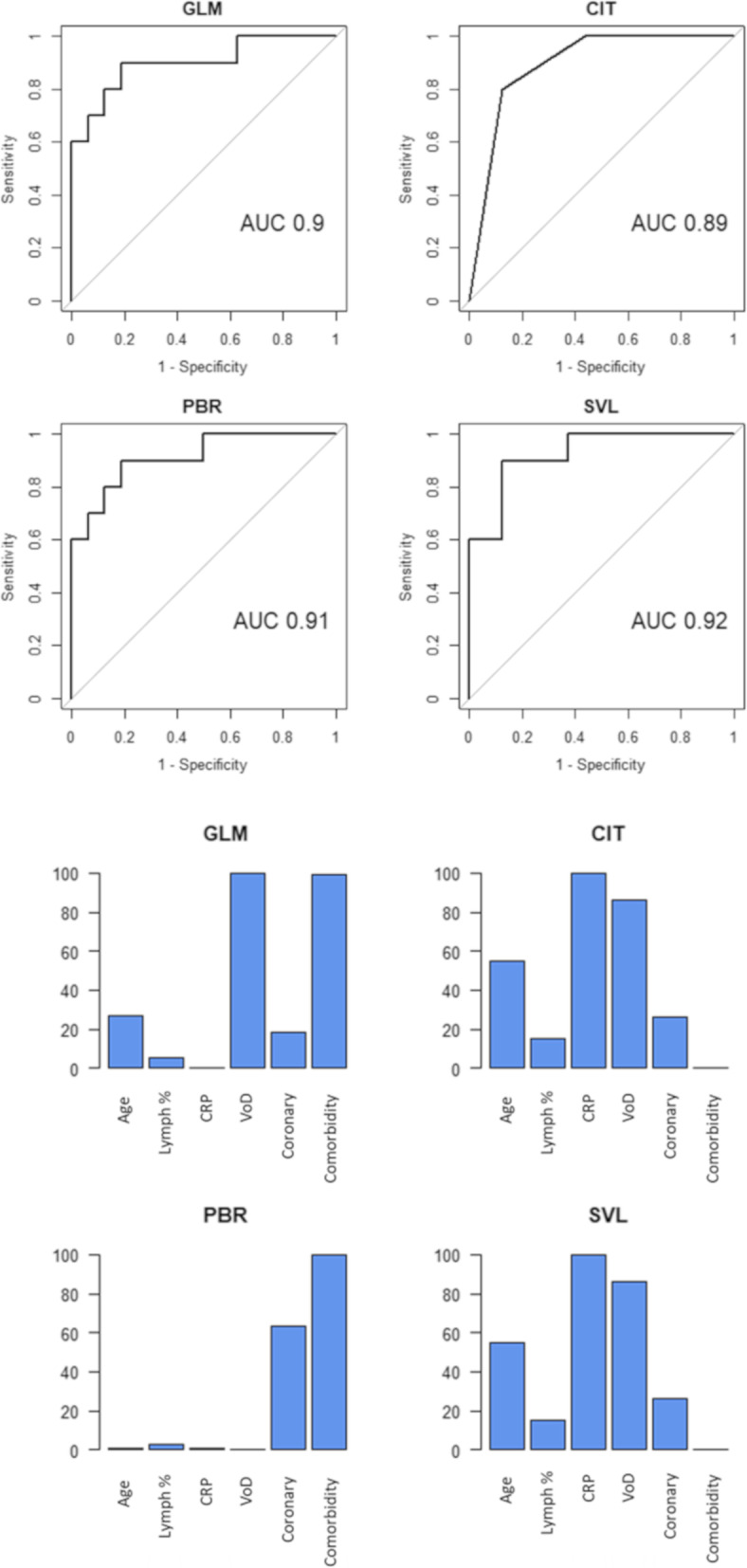
Shows receiver operating characteristic curve analysis of each model and the corresponding variable importance. AUC, Area under the curve; CIT, Conditional inference trees; CRP, C-reactive protein; GLM, Generalised linear model; Lymph %, Lymphocyte percentage; PBR, Penalised binomial regression; SVL, Support vector machine with linear kernel; VoD, Volume of disease

The repeated, cross-validated training was performed on the CIT, PBR, and SVL algorithms, whose parameters need tuning before a final model can be built. The training yielded a maximum AUC of 0.72 for CIT, 0.80 for PBR, and 0.82 for SVL. The parameters associated with those AUCs were used to re-train the algorithms, thus providing a final version for the models. The results for the final models (now including GLM) are reported in Table 3. CIT produced the lowest prediction accuracy (73.1%) among the algorithms tested, followed by PBR (80.8%) and GLM (84.6%), while the highest accuracy was found for SVL (88.5%). ROC curves for the models’ predictions are shown in Fig. 5. All the models explored here behave better than a random classifier (ROC curve close to the graph diagonal), although the AUC indicates better performance for the SVL algorithm (AUC 0.92) than for CIT (AUC 0.89), GLM (AUC 0.90), and PBR (AUC 0.91). The variable contribution for each algorithm is shown in Fig. 5. Overall, SVL resulted in the best performing model (Table 3). Table 4 shows the confusion matrix for the SVL prediction on the test set. Supplementary material shows ROC curves for the model’s predictions without the inclusion of VoD (Supplementary figure S1).

**Table 3:** Overall model performance

	AUC	Accuracy	Sensitivity	Specificity	PPV	NPV
Generalised linear model	0.90	0.85	0.80	0.87	0.80	0.87
Conditional inference trees	0.89	0.73	1.00	0.56	0.59	1.00
Penalised binomial regression	0.91	0.81	0.70	0.87	0.78	0.82
Support vector machine with linear kernel	0.92	0.88	0.90	0.87	0.82	0.93

*AUC* Area under the curve, *NPV* Negative predictive value, *PPV* Positive predictive value

**Table 4:** Confusion matrix for the support vector machine with linear kernel prediction on the testing set

		Observed	
		Favourable	Adverse
Predicted	Favourable	14	1
	Adverse	2	9

## Discussion

Our work aimed to quantify the burden of COVID-19 lung involvement using a fast, simple, and widely available tool, which can be found in most basic workstations for CT imaging post-processing. We investigated whether several clinical and laboratory features could estimate the VoD, and subsequently predicted the early outcomes of these patients.

Our results show that CT and clinical information predict short-term outcomes with high accuracy. Overall inflammatory burden, measured by quantitative CT data (*i.e.,* VoD at baseline CT) and C-reactive protein levels, were the most important variables for outcome prediction in COVID-19 patients. C-reactive protein is an accurate estimator of the systemic disease burden; however, it cannot pinpoint the site of disease. CT is precise in both locating and grading COVID-19 lung involvement, and a solid predictor of outcomes. Three out of 4 models heavily relied on VoD to classify patients as either having a favourable or an adverse outcome (Fig. 5). Despite the differences in performance, the prediction of CIT and SVL is based on a similar model structure, as suggested by the variable contribution. In fact, both CIT and SVL predictions are mainly built on serum levels of C-reactive protein, VoD, and age, with minor contributions from coronary calcification and lymphocyte percentage, and little to no contribution from chronic comorbidities. The prediction of PBR is mainly based on coronary calcification and presence of chronic comorbidities. VoD and chronic comorbidities provided the major contribution to GLM predictions.

Previous research assessed the role of CT in predicting COVID-19 outcomes. Yuan et al. [12] developed a CT score based on qualitative findings and achieved a sensitivity of 85.6% for predicting mortality. Colombi et al. [13] performed the first study that used quantitative CT parameters to predict clinical outcome and concluded that quantification of well-aerated lung provided higher accuracy in predicting severe outcome compared to clinical parameters alone. Our results are in line with those reported by these previous studies and suggest that CT may be an effective tool for the initial individual risk assessment. This is of exceptional importance primarily in those cases whose symptoms and overall general condition do not suggest severe lung disease. However, quantitative CT data alone are not enough to predict short-term outcomes. In this regard, we also analysed the influence of patient-related factors in the definition of early outcome. The most predictive patient-related factors of adverse outcome were age and the presence of a significant comorbidity.

The combination of CT, clinical, and laboratory findings can provide valuable information to direct toward a correct diagnosis while waiting for RT-PCR results. This was demonstrated by some works that showed a high sensitivity of CT in diagnosing COVID-19 in patients with respiratory symptoms [3, 4]. In the background of high disease prevalence, the relatively lower specificity of CT is mitigated by the low likelihood of alternative diagnosis. In our experience, during times of high disease prevalence, a patient presenting with pneumonia symptoms and with a CT showing bilateral peripheral/mixed GGO with or without concurrent consolidations and absent pleural effusion was highly suggestive of COVID-19.

Some studies correlated CT findings with the overall disease burden. Yang et al. [10] used a CT severity score based on qualitative and semi-quantitative features; they successfully discriminated mild from severe disease. Zhang et al. [11] reported similar results and concluded that some CT findings were more prevalent in the severe disease group. In another study [8], a deep learning model found significant differences in quantitative CT opacification parameters across different clinical types of COVID-19 patients. In our work, we used a less sophisticated method for quantification of lung opacities at CT. Still, we found a significant association between the VoD and systemic inflammation burden, measured by C-reactive protein. On the other hand, we did not find a significant association between the duration of symptoms at the time CT was performed and the VoD. To that regard, Pan et al. [24] found maximum lung involvement 10 days after the first symptom. Likewise, Wang et al. [25] concluded that most of the patients progress to acute respiratory distress syndrome in 12 days or less from the first symptom. These findings supported our decision in choosing a 10-day endpoint for observing clinical outcomes.

Our study had some limitations. First, the retrospective nature of this study makes it prone to selection bias. CT was performed in patients who sought medical attention despite restrictive quarantine measures, *i.e.,* the patients with mild symptoms did not come to our institution. Second, this was a single-centre study, and therefore the included cohort of patients may limit the generalizability of our observations. However, all models achieved high accuracy in previously unseen samples, which is an indicator of generalizability to external data. Third, another limitation was that we did not consider the interpersonal variability of total lung volumes. This issue can be addressed by providing the percentage of involved lung by calculating the VoD over the total lung volume. Yet, if not performed automatically, this may be a lengthy process and not suitable for clinical practice. Moreover, in our work we calculated COVID-19 lung involvement directly from the CT workstation; image transfer to open source third-party software tools may be difficult to perform in high-volume clinical settings. Fourth, we did not evaluate inter-rater agreement in VoD quantification. It is plausible that quantitative CT data may be less prone to interobserver variability than qualitative CT findings and thus more reliable in the prediction of short-term outcome in COVID-19 patients. Fifth, even though patients that did not require hospitalisation were instructed to return to our institution as soon as their symptoms worsened, a small fraction of individuals could have been lost in follow-up. Sixth, we did not correlate quantitative CT findings with other important clinical data (*e.g.,* hypertension, D-dimer, peripheral capillary oxygen saturation); unfortunately, these data were not available in all patients. We used CT surrogates for cardiovascular disease (aortic and coronary calcifications), which may limit the applicability of these results to the real world. Finally, another limitation is that we grouped all comorbidities in one feature. Yet, this allowed for statistical robustness considering the relatively low number of patients.

In conclusion, measuring the VoD in the lungs using a simple CT post-processing tool allows estimation of COVID-19 burden. The VoD was predicted by C-reactive protein levels and lymphocyte percentage. Clinical and laboratory information combined with quantitative CT data provided a prediction of short-term clinical outcomes in COVID-19 patients.

## Supplementary information

**Additional file 1: Supplementary figure S1.** ROC curves for the model’s predictions without the inclusion of VoD.

## Data Availability

Data will be made available on reasonable request.

## Abbreviations

AUC: Area under the curve
CIT: Conditional inference trees
COVID-19: Coronavirus disease 2019
GLM: Generalised linear model
GGO: Ground-glass opacities
PBR: Penalised binomial regression
ROC: Receiver operating characteristic
RT-PCR: Reverse transcription-polymerase chain reaction
SARS-CoV-2: Severe acute respiratory syndrome coronavirus 2
SVL: Support vector machine with linear kernel
VoD: Volume of disease
