# Supplementary figures and images for: Evaluation of novel coronavirus disease (COVID-19) using quantitative lung CT and clinical data: prediction of short-term outcome
Source: Eur Radiol Exp. 2020 Jun 26;4:39. doi: 10.1186/s41747-020-00167-0 (PMC7318726; doi:10.1186/s41747-020-00167-0)

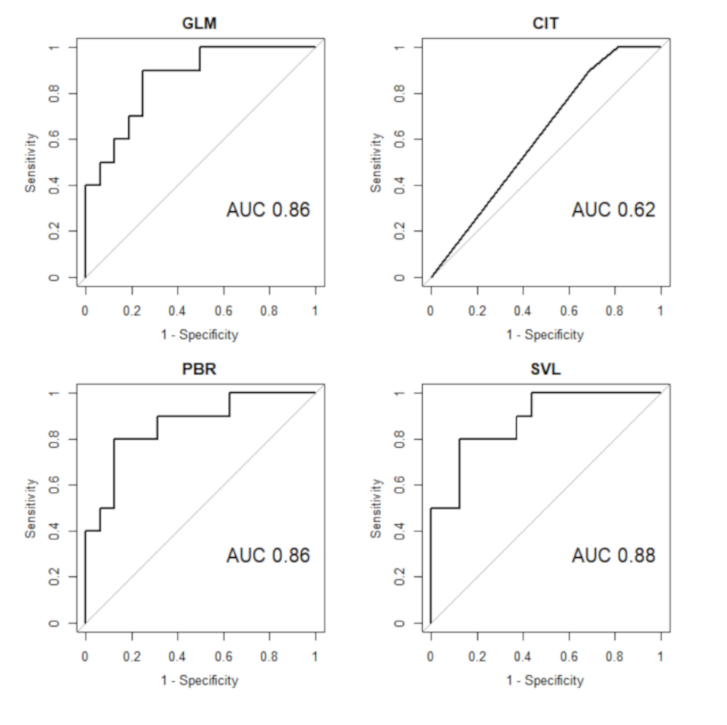


Supplementary figure S1

Supplement: Supplementary file 1 — Additional file 1: Supplementary figure S1. ROC curves for the model’s predictions without the inclusion of VoD. [file 41747_2020_167_MOESM1_ESM.docx]
